# Impact of Gender and Age on Hyperthermia-Induced Changes in Respiration of Liver Mitochondria

**DOI:** 10.3390/medicina54040062

**Published:** 2018-08-31

**Authors:** Giedrė Šilkūnienė, Rasa Žūkienė, Zita Naučienė, Laima Degutytė-Fomins, Vida Mildažienė

**Affiliations:** Department of Biochemistry, Faculty of Natural Sciences, Vytautas Magnus University, LT-44404 Kaunas, Lithuania; Giedre.Silkuniene@vdu.lt (G.Š.); Rasa.Zukiene@vdu.lt (R.Ž.); Zita.Nauciene@vdu.lt (Z.N.); Laimadf@gmail.com (L.D.-F.)

**Keywords:** age, gender, hyperthermia, liver mitochondria, reactive oxygen species, respiration

## Abstract

*Aim*: This study aimed to compare hyperthermia-induced changes in respiration and generation of reactive oxygen species (ROS) in liver mitochondria derived from animals of different gender and age. *Methods*: The effects of hyperthermia (40–47 °C) on oxidation of different substrates and ROS production were estimated in mitochondria isolated from the liver of male and female rats of the 1–1.5, 3–4, or 6–7 months age. *Results*: Gender-dependent differences in response of respiration to hyperthermia were the highest at 3–4 months of age, less so at 6–7 months of age, and only minor at juvenile age. Mild hyperthermia (40–42 °C) stimulated pyruvate + malate oxidation in mitochondria of females, but inhibited in mitochondria of males in the 3–4 month age group. The resistance of mitochondrial membrane to hyperthermia was the highest at 3–4 month males, and the lowest in the 6–7 month age group. Inhibition of glutamate + malate oxidation by hyperthermia was caused by thermal inactivation of glutamate dehydrogenase. ROS generation at 37 °C was higher at 1–1.5 month of age, but the increase in ROS generation with rise in temperature in this age group was the smallest, and the strongest in 6–7 month old animals of both genders. *Conclusions*: The response to hyperthermia varies during the first 6–7 months of life of experimental animals: stronger gender dependence is characteristic at 3–4 months of age, while mitochondria from 6–7 months animals are less resistant to hyperthermia.

## 1. Introduction

Hyperthermia (HT) is clinically used to treat a wide range of cancers including various types of abdominal, cervical, ovarian and breast cancers, neoplastic lesions of the head and neck, as well as regional tumors, such as melanoma skin cancer [1]. The efficiency of hyperthermal oncotherapy varies strongly in different types of cancer, but the molecular reasons underlying differences in response to HT of various cancer cells and normal tissues are not yet established [1]. The question of the possible contribution of gender and age factors to response to HT in various tissues types is unresolved, even though a body of evidence suggests that multiple functions can be remarkably influenced by sexual dimorphism and aging at different organization levels of the organism [2,3,4,5,6,7]. Nevertheless, male animals are still preferred in most experimental studies [8,9] leaving the female gender strongly underrepresented in biomedical research.

It has been recognized that the functions of healthy liver tissue exert remarkable gender dependence. In terms of gene expression, the liver is a very dimorphic organ—approximately 70% of genes have different expression levels in different genders [10]. Numerous studies have reported that the liver of females is more tolerant of stressful conditions (e.g., ischemia/reperfusion [11], hemorrhage/resuscitation [12], and alcoholic liver injury [13]), compared to that of males. Such findings imply that gender dependency may be characteristic in the response of liver tissue and cellular structures to HT.

Mitochondria are the key players in the cellular response to heat and HT-induced cell death via apoptotic pathway (see review [14]. Enhancement of ROS generation in mitochondria is involved in HT-induced cell injury and induction of apoptosis (reviewed by Slimen et al. [15]). Sexual dimorphism of mitochondrial function in a range of cells and tissues was reviewed recently [8,16], and numerous evidences were presented regarding the involvement of mitochondria in sex specific ageing and disease processes. However, the effects of HT have not yet been considered in such context. Data on sexual dimorphism in oxidative capacity of liver mitochondria are somewhat controversial [17,18,19,20,21]. It has been reported that HT of 45–47 °C induces a stronger increase in the membrane permeability of liver mitochondria isolated from female rats than that of males [22]. In this study we aimed to examine gender-dependent aspects of mitochondrial response to HT in more detail, including estimating induced changes in mitochondrial respiration, ROS production, and the dependence of these processes on animal age.

The aim of this study was to estimate the effect of incubation in febrile (40 °C) and supraphysiological (41–47 °C) temperature on respiration with pyruvate + malate (P+M) or glutamate + malate (G+M) in metabolic state 2 (V_2_), state 3 (V_3_) (according terminology of Chance and Williams [23]), and uncoupled state (V_CCCP_), in mitochondria isolated from the liver of male and female rats of different age (1–1.5, 3–4, 6–7 months). Most similar studies have compared very large (17–20 months) differences in age [24,25,26]. We intended to estimate age (but not ageing)-related differences in mitochondrial function, including the response to heat. Therefore, mitochondria from very young animals before puberty and two groups of sexually matured [27,28] but not senescent Wistar rats (both 3–4 and 6–7 months animal groups belong to the age interval widely used for biomedical research) were compared, so that the age difference among groups in this study was only several months. Since perturbations of mitochondrial respiration cause changes in ROS production, we also determined the effect of HT on ROS production in liver mitochondria derived from animals of different genders and age.

## 2. Materials and Methods

### 2.1. Experimental Animals

The experiments were carried out on mitochondria isolated from the liver of male and female Wistar rats. Permission No. 0155 was obtained from the Ethics Committee of the usage of laboratory animals at the Lithuanian State Food and Veterinary Service. The animals were acclimatized to 22 °C and a 12 h light-dark cycle (lights on at 08:00 h), had free access to water, and a standard chow diet. Three age groups of rats of both genders were used for the experiments—1–1.5 months, 3–4 months, and 6–7 months. Male and female animals weighed 71 ± 5 and 69 ± 8 g (1–1.5 months), 276 ± 21 and 204 ± 4 g (3–4 months); 470 ± 30 and 275 ± 16 g (6–7 months), respectively.

### 2.2. Isolation of Mitochondria

Mitochondria were isolated from livers of male and female Wistar rats belonging to three age groups (1–1.5, 3–4, and 6–7 months) as previously described [29]. Liver tissue was cut into small pieces and homogenized in a glass-teflon homogenizer. Homogenization medium contained 250 mM sucrose, 10 mM Tris, 3 mM EGTA and 2 mg/mL bovine serum albumin (BSA) (pH 7.7, 2 °C). The homogenate was centrifuged at 750× *g* for 5 min, and the supernatant was then centrifuged at 6800× *g* for 10 min. The mitochondrial pellet was resuspended in a suspension buffer containing 250 mM sucrose, 5 mM Tris-HCl (pH 7.3, 2 °C) and centrifuged again. The mitochondrial pellet was resuspended again in a suspension buffer and stored on ice. Protein content was determined by the modified biuret method [30].

### 2.3. Determination of the Dissolved Molecular Oxygen Concentration

The concentration of molecular oxygen dissolved in the assay medium at different temperatures (37–47 ± 0.1 °C) was determined polarographically using glucose oxidase catalyzed reaction between d-glucose and O_2_, while the pH of the medium was strictly controlled at each temperature (pH 7.2). The molar ratio coefficient of the reaction between d-glucose and O_2_ was defined in the medium with known concentration of dissolved oxygen at 37 °C.

### 2.4. Measurement of Mitochondrial Respiration

Mitochondrial respiration at different temperatures (37–47 ± 0.1 °C) was measured in a closed, stirred and thermostated 1.5 mL glass vessel equipped with Clark-type oxygen electrode. The incubation medium (IM) contained 20 mM Tris, 5 mM KH_2_PO_4_, 110 mM KCl, 50 mM creatine, 2.3 mM MgCl_2_, pH 7.2. Excess of creatine kinase (0.1 mg/mL) was added to maintain a steady state respiration. The experiments were performed using 5 mM pyruvate plus 5 mM malate (P+M) or 5 mM glutamate plus 5 mM malate (G+M) as oxidizable substrates. Mitochondria (1 mg protein/mL) were incubated in the assay medium with the respiratory substrate (state 2) for 3 min at 37, 40, 42, 45, or 47 °C before the state 3 respiration was initiated by addition of 1 mM ATP. The respiration rate in the uncoupled state was induced by addition of 0.5 µM carbonylcyanide m-chlorophenylhydrazone (CCCP) and 1 mM ATP. The rate in uncoupled state was denoted as V_CCCP_. The rates of mitochondrial respiration in state 2 (V_2_), state 3 (V_3_), and the respiratory control index (RCI, V_3_/V_2_) were defined according to the conventional terminology [23]. RCI was used to estimate the quality of isolated mitochondrial preparations and in our experiments RCI was about 5 with G+M.

### 2.5. Measurement of Pyruvate Dehydrogenase (PDH) and Glutamate Dehydrogenase (GDH) Activity in Liver Mitochondria

Activity of enzymes was measured spectrophotometrically at 340 nm detecting NADH oxidation rate for GDH activity by modified Ellis and Goldberg method [31], and NAD^+^ reduction rate for PDH activity by modified Hinman and Blass method [32]. The activity of enzymes was estimated in the 0.05% Triton X-100 lysates of mitochondria (0.2 mg protein/mL) at 37, 40, 42, 45, and 47 °C. Medium for GDH activity measurements contained 20 mM Tris, 120 mM KCl, 2 mM EGTA, 50 mM (NH_4_)_2_SO_4_, and pH was adjusted to 7.4 at all temperatures. GDH reaction was started by adding 0.1 mM NADH, 50 mM 2-oxoglutarate. IM without creatine was used for PDH activity measurements, pH was adjusted to 8.0 at all temperatures. PDH reaction was started by adding 2.5 mM NAD^+^, 0.1 mM CoA, 0.4 mM thiamine pyrophosphate, 5 mM pyruvate, 10^−6^ M rotenone. Measurement duration was 3 min at each of indicated temperature points.

### 2.6. Measurement of Mitochondrial ROS Production

As in the most of similar studies, the rate of ROS generation in mitochondria was estimated in state 2. Therefore, the rate of ROS generation was determined in state 2 using the incubation medium (IM) without creatine by a previously described method [33]. The rate of ROS generation was evaluated by an accumulated ROS amount after 30 min incubation at different temperatures ranging from 37 to 45 °C. After the incubation of 0.5 mg mitochondrial protein/mL with P+M or G+M, 5 μM Amplex Red (*N*-acetyl-3,7-dihydroxyphenoxazine) and 10 U/mL horseradish peroxidase, ROS amount was determined fluorimetrically using a thermostated fluorimeter “Tecan GENios Pro™” (Tecan Group Ltd., Menedorf, Switzerland). Measurements were carried out in 96-well plates at 535/590 ± 10 nm excitation/emission wavelengths. Calibration was performed by adding 5 μmol H_2_O_2_.

### 2.7. Statistical Analysis

Data are presented as means of 4–10 independent experiments ± standard error (s.e.m.). The means of each individual experiment with the same mitochondrial preparation were obtained repeating each measurement 3 times. Data distribution analysis performed by Shapiro-Wilk test (appropriate for small sample sizes) with significance value *p* > 0.05 showed that most of our data met the criteria for normal distribution. Statistical analysis was performed with Student’s *t*-test for comparison of two independent groups or one-way ANOVA, with Tukey post-hoc analysis for comparison of data changes in each temperature. The differences were assumed to be statistically significant at *p* < 0.05.

## 3. Results

**Figure 1:**
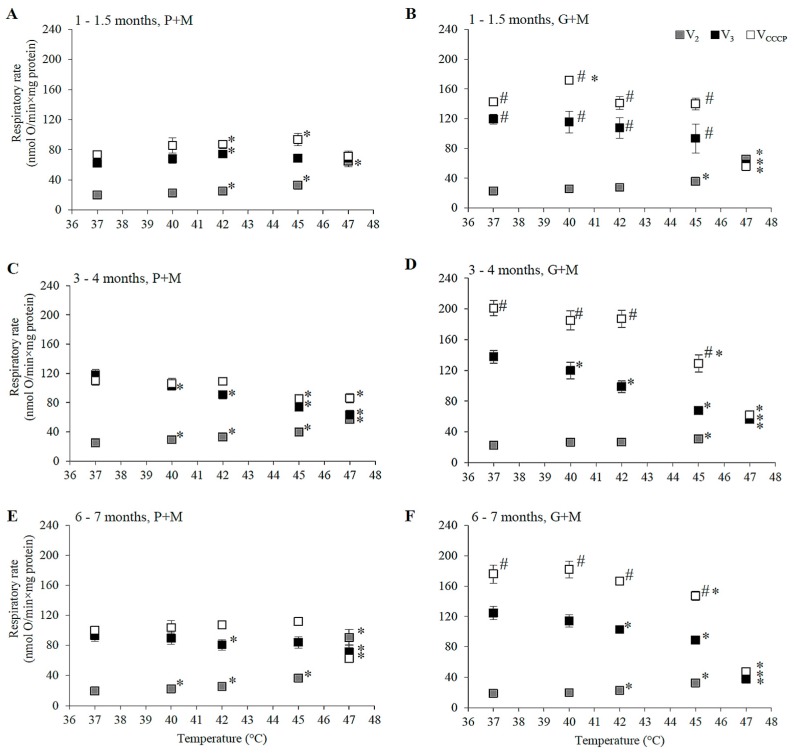
The dependence of state 2, state 3 and uncoupled respiration rates in isolated male rat liver mitochondria on the incubation temperature. (**A**,**C**,**E**) P+M oxidation, (**B**,**D**,**F**)—G+M oxidation; (**A**,**B**) 1–1.5 months, (**C**,**D**) 3–4 months, and (**E**,**F**) 6–7 month age group; State 2 (grey squares), state 3 (black squares), and uncoupled respiration (open squares) are presented in each panel. Values are means ± s.e.m., N = 4–10 independent mitochondrial preparations (error bars smaller than symbols are not shown). *—statistically significant (*p* < 0.05) difference as compared to 37 °C. #—statistically significant (*p* < 0.05) difference as compared to P+M oxidation.

**Figure 2:**
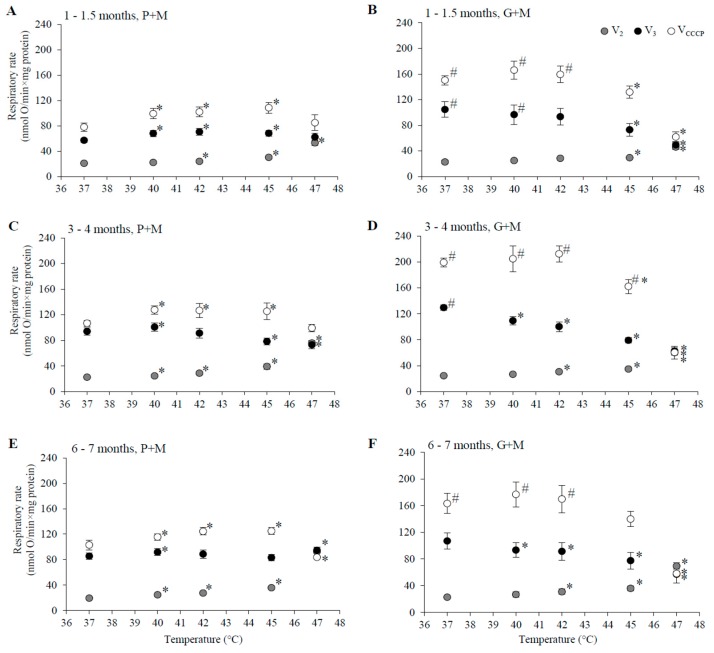
The dependence of state 2, state 3, and uncoupled respiration rates in isolated female rat liver mitochondria on the incubation temperature. (**A**,**C**,**E**) P+M oxidation, (**B**,**D**,**F**) G+M oxidation; (**A**,**B**) 1–1.5 months, (**C**,**D**) 3–4 months, and (**E**,**F**) 6–7 month age group; State 2 (grey circles), state 3 (black circles) and uncoupled respiration (open circles) are presented in each panel. Values are means ± s.e.m., N = 4–10 independent mitochondrial preparations (error bars smaller than symbols are not shown). *—statistically significant (*p* < 0.05) difference as compared to 37 °C. #—statistically significant (*p* < 0.05) difference as compared to P+M oxidation.

In this study we evaluated the impact of age and gender on the rates of P+M and G+M oxidation in different metabolic states (V_2_, V_3_, V_CCCP_) in liver mitochondria respiring under physiological (37 °C) and hyperthermia treatment conditions (40–47 °C). Respiratory rates were estimated in female and male rats of three age groups: 1–1.5, 3–4, and 6–7 months. The dependence of respiratory rate on incubation temperature in mitochondria of male rats is presented in Figure 1, and those for mitochondria of female rats—in Figure 2.

### 3.1. Gender and Age Dependence of the Respiratory Rates at 37 °C

The value of V_2_ at 37 °C was close to 20 nmol O·min^−1^·mg protein^−1^, independent of oxidizable substrate, gender, or age (Figure 1 and Figure 2). Mitochondrial respiration rate in state 3 (V_3_) at 37 °C for mitochondria oxidizing P+M was dependent on animal age. In young rats (1–1.5 months, Figure 1A and Figure 2A) V_3_ was similar in both genders and was approximately 1.5-fold lower as compared to the 3–4 month age group. The highest V_3_ values were characteristic in mitochondria from 3–4 months old males (Figure 1C). At this age V_3_ dependence on gender was remarkable—V_3_ of female liver mitochondria (Figure 2C) at 37 °C was 20% lower (94 ± 6 nmol O·min^−1^·mg protein^−1^) than that of males (118 ± 7 nmol O·min^−1^·mg protein^−1^).

However, there was no difference in V_3_ between mitochondria of males (Figure 1E) and females (Figure 2E) in the 6–7 month age group. V_3_ in this group of males was 19% lower as compared to 3–4 months, but V_3_ of females was similar as that of 3–4 month old animals. The rate of uncoupled respiration (V_CCCP_) at 37 °C in mitochondria of males did not differ from V_3_ in all analyzed age groups (Figure 1A,C,E), while in mitochondria of females V_CCCP_ exceeded V_3_ values by 13–30% depending on the age group (Figure 2A,C,E).

The rates of respiration in mitochondria oxidizing G+M in state 3 under all experimental conditions were higher in comparison to the corresponding rates with P+M (Figure 1 and Figure 2). This difference was slightly more obvious in mitochondria isolated from males: V_CCCP_ values were from 40 to 65%, V_3_ values—from 13 to 48% higher as compared with the same values with P+M, whereas in female mitochondria these values ranged from 38–47% and from 19–42%, respectively. The age dependency of G+M oxidation was similar in mitochondria of both genders: V_3_ and V_CCCP_ were from 9–29% lower in 1–1.5 month and 6–7 months groups in comparison to the 3–4 month group. Gender-dependent differences in mitochondrial respiration rates with G+M were not statistically significant.

### 3.2. Effects of HT on Respiration of Mitochondria Oxidizing P+M

HT induced changes in the respiration rate (V_2_, V_3_, and V_CCCP_) were compared in liver mitochondria of males and females by subjecting mitochondria to different incubation temperatures above physiological temperature (37 °C) ranging from 40 to 47 °C. The changes in the respiratory rate that were observed are shown in Figure 1 and Figure 2, and statistically significant HT effects (in percentage of the respective control rates at 37 °C) for each gender and age group are presented for comparison in Table 1.

HT-induced progressive increase in V_2_ was characteristic for all experimental groups indicating an increase in permeability of the inner membrane of liver mitochondria, supporting earlier reports by other authors [22,34]. A statistically significant increase in V_2_ in P+M oxidizing mitochondria started at a lower temperature (40 °C) in males and females of older groups (3–4 and 6–7 months), while the uncoupling effect in mitochondria from 1–1.5 month age group became evident only at 42 °C. The membrane was more resistant to heating above 42 °C in the young female group, in comparison to the male group, while the opposite was observed for the 3–4 month age group. However, at higher temperatures (45 and 47 °C) the membrane of mitochondria from 3–4 month old males was more resistant to HT than all other groups. In contrast, membrane barrier function in mitochondria of animals of both genders in the 6–7 months age group was most severely compromised by HT at higher temperatures (V_2_ increase at 47 °C in 6–7 month old males was 2.8 larger than in 3–4 month old males, the same difference for females was 1.4-fold). There were no clear gender-dependent differences in HT-induced V_2_ changes in animals 6–7 months of age. For all experimental groups V_2_ increased so strongly that it overlapped with V_3_ (Figure 1 and Figure 2) at 47 °C, indicating complete uncoupling of oxidative phosphorylation.

State 3 and uncoupled respiration with P+M in young animals was stimulated or not affected by HT, and stimulation was more prominent in the female group (Figure 1A and Figure 2A). However, HT caused a decrease in V_3_ and V_CCCP_ in male animals in the 3–4 month old group, and the inhibition of V_3_ was twice as strong as the inhibition of V_CCCP_ (Figure 1C and Figure 2C). Contrary to inhibition in the male group, V_CCCP_ was stimulated by HT (at 40–45 °C) in mitochondria isolated from female rats of the same age, while V_3_ was stimulated at 40 °C, and inhibited to a smaller extent compared to V_3_ inhibition in male mitochondria at 45 and 47 °C.

Special attention should be paid to the comparison of respiration rates V_3_ between genders in the 3–4 month age group at normal, fever, and supra-physiological HT temperatures. Respiration in females at a normal body temperature (37 °C) was 29% lower than that of males, however under fever conditions mitochondria of female liver respired at the same rate as those of males. The rise in temperature above the physiological (fever-range) HT (i.e., up to 41–43 °C) significantly inhibited V_3_ only in male mitochondria, while V_3_ in mitochondria of females did not differ from V_3_ at 37 °C. V_3_ in females was 17% lower as compared to 37 °C only at 45 °C (37% in males, Table 1). The effects of HT on V_3_ and V_CCCP_ in mitochondria from males and females 6–7 months of age had similar trends, but were less pronounced in comparison to the 3–4 month age group (Figure 1E and Figure 2E).

### 3.3. Effects of HT on Respiration in Mitochondria Oxidizing G+M

The ability of HT to uncouple oxidative phosphorylation was obvious in mitochondria respiring with G+M (Figure 1B,D,F and Figure 2B,D,F, Table 1) similarly as it was with P+M (Figure 1A,C,E and Figure 2A,C,E, Table 1), but the extent of induced changes in V_2_ was dependent on the substrate—larger changes of V_2_ were observed in mitochondria respiring with P+M in comparison to G+M in all groups (Table 1). A statistically significant increase in V_2_ with G+M was registered only above 42 °C (Table 1). The uncoupling effect was stronger in male mitochondria as compared to female mitochondria in the 1–1.5 month age group, but gender dependence was less evident in older animals.

**Figure 3:**
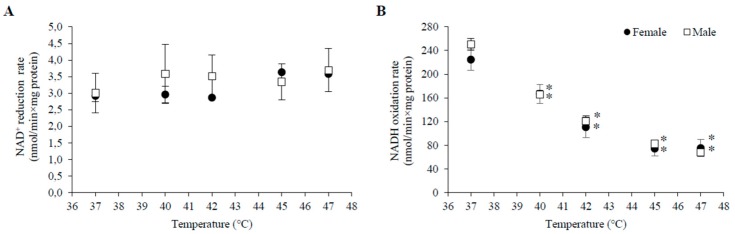
PDH and GDH activity in lysates of rat male and female mitochondria at different incubation temperatures. Male rat mitochondria—open squares; female rat mitochondria—black circles; (**A**) dependence of PDH complex activity (NAD^+^ reduction rate) on incubation temperature; (**B**) dependence of GDH activity (NADH oxidation rate) on incubation temperature. Values are means ± s.e.m. for N = 6 independent mitochondrial preparations. *—statistically significant (*p* < 0.05) difference as compared to 37 °C.

HT induced a decrease in V_3_ with G+M in all experimental groups. Statistically significant changes started at a lower temperature (40 °C) in both male and female mitochondria in 3–4 month old animals in comparison to 6–7 months (42 °C) and 1–1.5 months (45 °C for females, 47 °C—for males) old animals (Table 1). The extent of V_3_ inhibition increased with rising temperatures and reached up to 50–70% at 47 °C. HT also substantially inhibited V_CCCP_ in mitochondria of all groups, with the only exception being an increase in V_CCCP_ at 40 °C in mitochondria of males and females in the 1–1.5 month age group (Figure 1B and Figure 2B). The substrate-dependent difference in HT-induced effects was especially large in female mitochondria of all age groups (Table 1). Stronger negative effects on state 3 and uncoupled respiration with G+M in comparison to P+M imply that the enzymatic system of glutamate oxidation is more sensitive to HT than that of pyruvate. This indicated that GDH or glutamate transport can be inactivated by HT. To test this, we compared the activity of GDH and PDH in lysates of mitochondria isolated from male and female rat liver (3–4 months of age) at different incubation temperatures (37, 40, 42, 45, 47 °C). The obtained results demonstrate an obvious difference in thermal sensitivity of GDH and PDH (Figure 3). However, the activity of both enzymes at different temperatures was not gender-dependent.

The mean values of PHD activity were 4–5% higher at 40 and 42 °C, and 10–14% lower at 45 °C compared to the activity at 37 °C, but these differences were not statistically significant, indicating that enzymatic activity is not affected by temperature (Figure 3A). In comparison to PDH activity at 37 °C, the mean values were by 11–23% higher at 40–47 °C temperature range in male mitochondria, and by 23–25% higher at 45–47 °C in female mitochondria. However, these differences were not statistically significant, therefore we conclude that PDH activity was not affected by temperature (Figure 3A).

The obtained results show that opposite effects on respiratory subsystem activity (V_CCCP_) with P+M in female and male mitochondria hardly can be explained by effects on PDH activity. Thus, reasons for gender dependent response of this subsystem to HT may be related to other enzymes or transporters, e.g., pyruvate carrier.

In contrast to PDH, GDH was very sensitive to moderate heating—it was inactivated by approximately 50% and 70% after 3 min incubation at 42 and 47 °C, respectively (Figure 3B). Due to the stronger inhibition of the respiratory subsystem, the uncoupling effect on V_2_ with G+M was much lower in 45–47 °C, compared to P+M oxidation in mitochondria from females but not from males. Inhibition of V_3_ in all groups, except 1–1.5 month old females, was observed at lower temperatures than inhibition of V_CCCP_, indicating possible sensitivity of the phosphorylation subsystem to HT, similarly as in the experiments with P+M. Moreover, complete uncoupling of oxidative phosphorylation was induced at 47 °C for both substrates (Figure 1 and Figure 2).

### 3.4. Effects of HT on Mitochondrial ROS Generation

**Figure 4:**
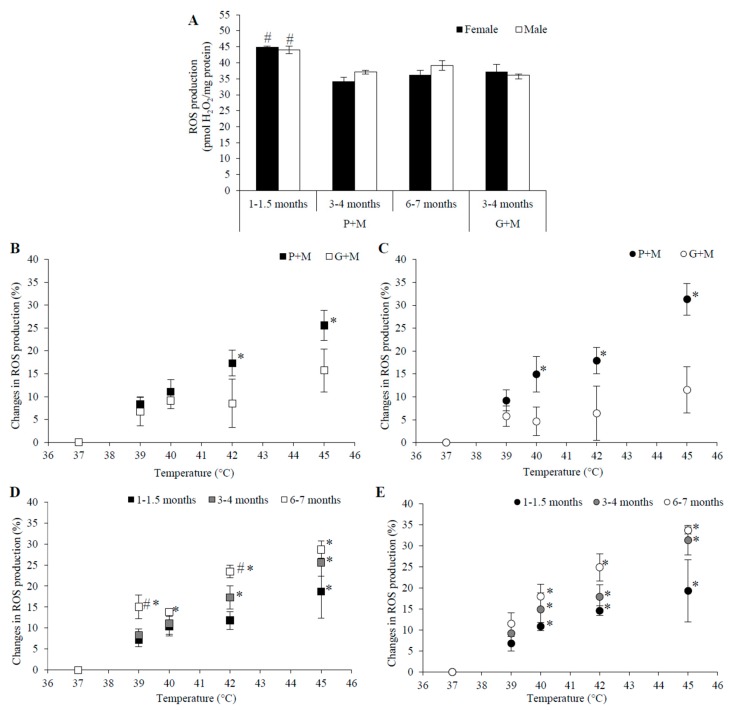
The dependence of mitochondrial ROS generation on rat age and gender. (**A**) ROS generation at 37 °C using different substrates—P+M and G+M, white columns—male rat mitochondria; black columns—female rat mitochondria; (**B**,**C**) the dependence of changes in ROS generation on incubation temperature in mitochondria isolated from 3–4 month old males (**B**) and females (**C**) rat liver, black symbols—substrate P+M, open symbols—substrate G+M; (**D**,**E**)—the dependence of changes in ROS generation on incubation temperature in male liver mitochondria isolated from male (**D**) and female (**E**) rats of different ages, substrate—P + M, black symbols—1–1.5 months, grey symbols—3–4 months, open symbols—6–7 months. Values are means ± s.e.m. for N = 4–8 independent mitochondrial preparations. #—statistically significant (*p* < 0.05) age effect as compared to the 3–4 month age group, *—statistically significant (*p* < 0.05) difference as compared to 37 °C.

There was no significant difference between ROS generation at 37 °C with P+M or G+M as the respiratory substrates in either male or female mitochondria in the 3–4 month age group (Figure 4A). The rate of ROS production at 37 °C was the same in P+M oxidizing mitochondria from rats in 3–4 and 6–7 month age groups. However, ROS generation was higher for both genders in the young age group (1–1.5 months), as compared to that for older animals—32% and 24% higher in female mitochondria, and 19% and 13% higher in male mitochondria in 3–4 and 6–7 month age groups, respectively.

The effects of HT on ROS production in mitochondria oxidizing different substrates were compared in mitochondria of animals of both genders from the 3–4 months age group (Figure 4B,C). The results revealed that ROS generation in mitochondria using P+M was stimulated by 17% at 42 °C (in mitochondria of both males and females), but the respective changes in mitochondria oxidizing G+M were not statistically significant. Temperature rise to 45 °C increased ROS production with P+M by 26% in male and 31% in female mitochondria, while the effects were much smaller with G+M (16% and 12%).

The obtained results demonstrate that even though G+M oxidation is characterized by much higher V_3_ and V_CCCP_ than oxidation of P+M, the rates of ROS generation at 37 °C are similar with both substrates (Figure 4A). The rise of temperature from 37 to 45 °C induced progressive increase in ROS production rate with P+M, but this effect was much smaller with G+M. This finding may also be explained by the strong negative effects of HT on the G+M oxidative system (Table 1) due to GDH inhibition (Figure 3B). ROS generation with G+M is stimulated by HT at 45 °C, i.e., even under conditions when the V_3_ and V_CCCP_ is inhibited (Table 1). Independently of age and gender, the rise of temperature was associated with the progressive increase in mitochondrial ROS generation when P+M was used as the substrate for respiration (Figure 4D,E). The response to HT was clearly dependent on animal age. Although ROS production in mitochondria from young animals was highest at 37 °C (Figure 4A), in both genders HT stimulated it less in comparison to other age groups. Stimulation was more pronounced in female mitochondria, where a statistically significant relative increase was achieved starting at 40 °C temperature, whereas for male mitochondria a statistically significant change was obtained at 45 °C. That might be explained by stronger activation of the respiratory chain in P+M oxidizing mitochondria of young female animals (Figure 1A and Figure 2A and Table 1).

HT induced the largest increase of ROS production in the older animal group (6–7 months), and this increase at 45 °C was significantly larger in mitochondria from female (34%) as compared to male (29%) animals. Gender-dependent differences may be related to stronger activation of the respiratory chain in mitochondria from female animals; however, the age-dependent differences in ROS production do not correlate with HT-induced effects on mitochondrial respiration, e.g., ROS generation was increased progressively in P+M oxidizing mitochondria in both genders in the 3–4 month age group, despite the observed opposite effects of HT on respiration in uncoupled state of male (inhibition) and female (stimulation) mitochondria.

## 4. Discussion

In contrast to findings of other authors [18,20,21,35], we did not obtain larger rates of respiration in mitochondria isolated from female liver in comparison to male liver in the absence of HT. In this respect our results were similar as those reported by Lash et al. [17]. Controversial results can at least in part be explained by a large variation of experimental conditions (animal age, substrates for mitochondrial respiration, incubation temperatures, etc.) used in different studies. In our experiments, mitochondrial respiration with P+M (but not G+M) was highest in both genders in the 3–4 month age group, which is most commonly used in biomedical research. Mitochondrial respiration (only with P+M in state 3, but not with G+M) was 20% higher in male mitochondria as compared to female mitochondria in this group only (Figure 1C and Figure 2C). Such a finding indicates that even such a small difference in age (as two months) in rats of both genders has an impact on the oxygen consumption rate and gender-dependence. The absence of gender-dependence for state 3 respiration in animals before puberty (1–1.5 months old) can be explained by smaller differences in hormonal regulation in comparison to mature animals (3–4 months old); however, such absence was unexpected in 6–7 month old animals.

HT induced progressive increase in state 2 respiration with both substrates in all experimental groups. Oxygen consumption in this metabolic state is almost exclusively controlled by the proton leak [36,37]; therefore, the V_2_ increase shows an increase in the proton leak of the inner mitochondrial membrane. The results obtained in temperatures between 37 and 47 °C (Figure 1 and Figure 2, Table 1) provide experimental evidence that thermal sensitivity of the inner mitochondrial membrane is dependent on age, gender, and the respiratory substrate. It is noteworthy that the effect of HT on the membrane barrier function was the smallest in the experimental group that is most commonly used in experimental research (3–4 months old male rats). The membrane of mitochondria from young rats was more resistant to heating at lower (40–42 °C) temperatures, but the membrane of the 3–4 month age group was the most resistant in a higher range (45–47 °C), while that of the 6–7 month old rats was the most sensitive to heating. HT-induced increase in the mitochondrial membrane leak was smaller when mitochondria oxidized G+M as compared to P+M. Since the membrane leak is strongly dependent on membrane potential [38], substrate-dependent differences in the effects on the proton leak can be explained by the thermal inhibition of GDH (Figure 3A) leading to a lower membrane potential in state 2, generated by the respiratory chain in mitochondria oxidizing G+M. Therefore, a statistically significant increase in V_2_ with G+M in all groups was observed at higher temperatures in comparison to P+M (Table 1). The uncoupling effect was stronger in male mitochondria as compared to female mitochondria in the 1–1.5 month age group, but gender dependence was less evident in older animals. Complete uncoupling of oxidative phosphorylation was induced at 47 °C in all experimental animal groups with both substrates (Figure 1 and Figure 2).

The response patterns of mitochondrial respiration to HT in state 3 and in the uncoupled state were also dependent on rat age, gender, and the substrate used for respiration. Substantial activation of the respiratory chain by HT (indicated by an increase in V_3_ and V_CCCP—_more pronounced in female mitochondria) was observed in mitochondria of 1–1.5 month-old rats oxidizing P+M (not G+M). A much stronger gender dependence, and even opposite HT-induced effects on V_CCCP_ with P+M was obtained in two older age groups, where the respiration rate was inhibited in male mitochondria but stimulated in female mitochondria. It is noteworthy that V_3_ values in the temperature range of 37–47 °C were similar as V_CCCP_ values in male mitochondria (3–4 months group); however, V_CCCP_ was higher than V_3_ in female mitochondria (Figure 1C and Figure 2C). This indicates a higher contribution of the phosphorylation subsystem to the control of respiratory flux in female rather than male mitochondria. The fact that V_3_ decreases with an increase of temperature from 42 to 45 °C in female mitochondria (Table 1), and V_CCCP_ increases as compared to 37 °C, shows that the respiratory subsystem in female mitochondria is activated at this temperature range, but the phosphorylation subsystem is inhibited. Inhibition of phosphorylation possibly goes along with the inhibition of the respiratory chain in mitochondria of the same age male rats because V_3_ is inhibited more strongly than V_CCCP_ in the whole temperature range from 40 to 47 °C.

We explain the substrate-related differences and the inhibition of state 3 and uncoupled respiration with G+M observed in all experimental groups by a strong thermal inactivation of GDH (Figure 3B). Rapid thermal inactivation of GDH purified from liver mitochondria has been reported previously [39], but the relevance of such observations for HT-induced effects on mitochondrial respiration or survival of cancer cells has not been considered. The possible importance of GDH inactivation upon HT cannot be overlooked in the light of recent findings concerning the central function of mitochondria and GDH in biosynthetic metabolism of rapidly growing “glutamine addicted” cancer cells [40,41]. It has been suggested that blocking glutamine entry into the TCA cycle might be one of the potential therapeutic approaches in cancer [42]. GDH regulates glutaminolysis, ammonia recycling and redox-balance in cancer cells, therefore it confers a proliferative advantage to cancer cells and tumor growth, ensures cancer cell survival under oxidative stress and is a potential anticancer target [43,44]. In the context of such findings, rapid inhibition of GDH upon HT should be strongly reconsidered in the context of the molecular mechanisms of tumor cell response to HT.

The comparison of HT induced changes in ROS generation in liver mitochondria isolated from rats of different gender and age has revealed that HT induced progressive increase in ROS generation in the whole range of studied temperatures from 37–45 °C in all experimental groups (Figure 4D,E). However, this effect was much smaller with G+M, possibly due to GDH inhibition. ROS generation in heart mitochondria has been reported to reach a maximum at 40 °C and decrease with further rise in temperature under similar conditions, possibly due to an abrupt increase in the membrane leak (decrease in membrane potential) at 42 °C temperature and above [45]. Uncoupling of liver mitochondria by heat reported here was continuous and more moderate in comparison to heart mitochondria. Stimulation of ROS production by HT was slightly stronger in female compared to male mitochondria, at least for animals in 3–4 and 6–7 month age groups.

We report obvious age-dependent differences in ROS generation in response to HT, even though the age interval between studied animal groups was only several months. At 37 °C temperature ROS generation was slightly higher in liver mitochondria from 1–1.5 month old animals in comparison to older animals, in agreement with the results obtained concerning superoxide production in the liver of 1.5 and 3 month old rats [46]. Mitochondrial respiration rates V_3_ and V_CCCP_ were lower in this group in comparison to rates observed in two other age groups, indicating that more rapid mitochondrial ROS generation in young animals may be not be related to the activity of respiration, but rather to the less adequate capacity of antioxidant defense. The activity of the most important antioxidative enzymes is strongly and progressively induced in time during the neonatal age of one month [47], and even in the age of 1–1.5 months these activities may still be below the level achieved in 3–6 months old rats. However, such an explanation does not help to understand why the stimulation of ROS production by HT was progressively stronger with increasing animal age (Figure 4D,E). Most likely, that reflects certain age-dependent differences in HT-induced redox changes of ROS-producing sites in the mitochondrial respiratory chain, leading to a faster passage of single electrons to oxygen.

## 5. Conclusions

The obtained data reveal that mitochondrial respiration, ROS production, and gender dependency of the response to HT vary substantially during the first 6–7 months of experimental animal life. Impact of gender was less obvious in juvenile animals (1–1.5 months old), only resistance of the membrane leak to HT in female mitochondria was higher while in male mitochondria—lower as compared with the same gender at 3–4 months of age. Despite small age difference between two other groups, they differed by many parameters characterizing functional activity of liver mitochondria, including stronger gender dependence at 3–4 months of age or higher vulnerability in response to HT (stronger uncoupling and increase in ROS production) in the 6–7 months age group. We showed that strong thermal inactivation of GDH is responsible for the observed inhibition of respiration by HT in liver mitochondria oxidizing G+M.

Both three and six months old rats are commonly used as the reference groups in experimental research, including studies on effects of HT. Therefore, the reported differences may be important for solving some inconsistencies in the obtained data or helpful for standardizing biomedical research.

## Figures and Tables

**Table 1:** The effects of hyperthermia on state 2, state 3, and uncoupled respiration rate in isolated rat liver mitochondria.

Age	Substrate, Metabolic State	Difference of Respiratory Rate (%) as Compared to 37 °C (*p* < 0.05)							
		Temperature, °C							
		40	42	45	47	40	42	45	47
		Male				Female			
1–1.5 months	P+M								
	V_2_	-	+25	+65	+201	-	+13	+43	+162
	V_3_	-	+19	-	-	+17	+22	+17	-
	V_CCCP_	-	+18	+27	-	+27	+31	+40	-
	G+M								
	V_2_	-	-	+57	+187	-	-	+26	+100
	V_3_	-	-	-	−51	-	-	−30	−52
	V_CCCP_	+21	-	-	−61	+11	-	−12	−59
3–4 months	P+M								
	V_2_	+16	+28	+60	+132	+8	+27	+77	+241
	V_3_	−14	−23	−37	−46	+8		−17	−21
	V_CCCP_	-	-	−20	−21	+20	+25	+24	-
	G+M								
	V_2_	-	-	+35	+143	-	+25	+48	+150
	V_3_	-13	−28	−50	−58	−16	−22	−39	−51
	V_CCCP_	-	-	−36	−69	-	-	−20	−65
6–7 months	P+M								
	V_2_	+16	+32	+89	+373	+27	+31	+46	+374
	V_3_	-	−13	-	−24	+8	-	-	-
	V_CCCP_				−36	+13	+22	+22	
	G+M								
	V_2_	-	+22	+76	+132	-	+35	+56	+187
	V_3_	-	−18	−29	−70	−13	−15	−28	−47
	V_CCCP_	-	-	−16	−70	-	-	-	−64

-, statistically not significant difference.

## References

[1] Mallory M., Gogineni E., Jones G.C., Greer L., Simone C.B. (2016). Therapeutic hyperthermia: The old, the new, and the upcoming. Crit. Rev. Oncol. Hematol..

[2] Guevara R., Gianotti M., Oliver J., Roca P. (2011). Age and sex-related changes in rat brain mitochondrial oxidative status. Exp. Gerontol..

[3] Colom B., Oliver J., Garcia-Palmer F.J. (2015). Sexual Dimorphism in the Alterations of Cardiac Muscle Mitochondrial Bioenergetics Associated to the Ageing Process. J. Gerontol. A Biol. Sci. Med. Sci..

[4] Lucas R.A., Sarma S., Schlader Z.J., Pearson J., Crandall C.G. (2015). Age-related changes to cardiac systolic and diastolic function during whole-body passive hyperthermia. Exp. Physiol..

[5] Sholomskas L.M., Roche K.L., Bloomer S.A. (2015). Aging impairs induction of redox factor-1 after heat stress: A potential mechanism for heat-induced liver injury. Int. J. Physiol. Pathophysiol. Pharmacol..

[6] Ratnu V.S., Emami M.R., Bredy T.W. (2017). Genetic and epigenetic factors underlying sex differences in the regulation of gene expression in the brain. J. Neurosci. Res..

[7] Valencak T.G., Osterrieder A., Schulz T.J. (2017). Sex matters: The effects of biological sex on adipose tissue biology and energy metabolism. Redox Biol..

[8] Demarest T.G., McCarthy M.M. (2015). Sex differences in mitochondrial (dys)function: Implications for neuroprotection. J. Bioenerg. Biomembr..

[9] Clayton J.A., Collins F.S. (2014). Policy: NIH to balance sex in cell and animal studies. Nature.

[10] Yang X., Schadt E.E., Wang S., Wang H., Arnold A.P., Ingram-Drake L., Drake T.A., Lusis A.J. (2006). Tissue-specific expression and regulation of sexually dimorphic genes in mice. Genome Res..

[11] Harada H., Pavlick K.P., Hines I.N., Hoffman J.M., Bharwani S., Gray L., Wolf R.E., Grisham M.B. (2001). Selected contribution: Effects of gender on reduced-size liver ischemia and reperfusion injury. J. Appl. Physiol..

[12] Jarrar D., Wang P., Cioffi W.G., Bland K.I., Chaudry I.H. (2000). The female reproductive cycle is an important variable in the response to trauma-hemorrhage. Am. J. Physiol. Heart Circ. Physiol..

[13] Colantoni A., Idilman R., De Maria N., La Paglia N., Belmonte J., Wezeman F., Emanuele N., Van Thiel D.H., Kovacs E.J., Emanuele M.A. (2003). Hepatic apoptosis and proliferation in male and female rats fed alcohol: Role of cytokines. Alcohol. Clin. Exp. Res..

[14] Ahmed K., Tabuchi Y., Kondo T. (2015). Hyperthermia: An effective strategy to induce apoptosis in cancer cells. Apoptosis.

[15] Slimen I.B., Najar T., Ghram A., Dabbebi H., Ben Mrad M., Abdrabbah M. (2014). Reactive oxygen species, heat stress and oxidative-induced mitochondrial damage. A review. Int. J. Hyperth..

[16] Ventura-Clapier R., Moulin M., Piquereau J., Lemaire C., Mericskay M., Veksler V., Garnier A. (2017). Mitochondria: A central target for sex differences in pathologies. Clin. Sci..

[17] Lash L.H., Qian W., Putt D.A., Hueni S.E., Elfarra A.A., Krause R.J., Parker J.C. (2001). Renal and hepatic toxicity of trichloroethylene and its glutathione-derived metabolites in rats and mice: Sex-, species-, and tissue-dependent differences. J. Pharmacol. Exp. Ther..

[18] Valle A., Guevara R., García-Palmer F.J., Roca P., Oliver J. (2007). Sexual dimorphism in liver mitochondrial oxidative capacity is conserved under caloric restriction conditions. Am. J. Physiol. Cell Physiol..

[19] Catala-Niell A., Estrany M.E., Proenza A.M., Gianotti M., Llado I. (2008). Skeletal muscle and liver oxidative metabolism in response to a voluntary isocaloric intake of a high fat diet in male and female rats. Cell Physiol. Biochem..

[20] Nadal-Casellas A., Amengual-Cladera E., Proenza A.M., Llado I., Gianotti M. (2010). Long-term high-fat-diet feeding impairs mitochondrial biogenesis in liver of male and female rats. Cell Physiol. Biochem..

[21] Chweih H., Castilho R.F., Figueira T.R. (2015). Tissue and sex specificities in Ca^2+^ handling by isolated mitochondria in conditions avoiding the permeability transition. Exp. Physiol..

[22] Nauciene Z., Zukiene R., Degutyte-Fomins L., Mildaziene V. (2012). Mitochondrial membrane barrier function as a target of hyperthermia. Medicina.

[23] Chance B., Williams G.R. (1955). Respiratory enzymes in oxidative phosphorylation. I. Kinetics of oxygen utilization. J. Biol. Chem..

[24] Stocco D.M., Cascarano J., Wilson M.A. (1977). Quantitation of mitochondrial DNA, RNA, and protein in starved and starved-refed rat liver. J. Cell Physiol..

[25] Tummino P.J., Gafni A. (1991). A comparative study of succinate-supported respiration and ATP/ADP translocation in liver mitochondria from adult and old rats. Mech. Ageing Dev..

[26] Sastre J., Pallardo F.V., Pla R., Pellin A., Juan G., O’Connor J.E., Estrela J.M., Miquel J., Viña J. (1996). Aging of the liver: Age-associated mitochondrial damage in intact hepatocytes. Hepatology.

[27] Robb G.W., Amann R.P., Killian G.J. (1978). Daily sperm production and epididymal sperm reserves of pubertal and adult rats. J. Reprod. Fertil..

[28] Goldman J.M., Laws S.C., Balchak S.K., Cooper R.L., Kavlock R.J. (2000). Endocrine-disrupting chemicals: Prepubertal exposures and effects on sexual maturation and thyroid activity in the female rat. A focus on the EDSTAC recommendations. Crit. Rev. Toxicol..

[29] Zukiene R., Nauciene Z., Silkuniene G., Vanagas T., Gulbinas A., Zimkus A., Mildažienė V. (2017). Contribution of mitochondria to injury of hepatocytes and liver tissue by hyperthermia. Medicina.

[30] Gornal A.G., Bardawill C.J., David M.M. (1949). Determination of serum protein by means of the burette reaction. J. Biol. Chem..

[31] Ellis G., Goldberg D.M. (1972). Optimal conditions for the kinetic assay of serum glutamate dehydrogenase activity at 37 degrees C. Clin. Chem..

[32] Hinman L.M., Blass J.P. (1981). An NADH-linked spectrophotometric assay for pyruvate dehydrogenase complex in crude tissue homogenates. J. Biol. Chem..

[33] Starkov A.A. (2010). Measurement of mitochondrial ROS production. Methods Mol. Biol..

[34] Willis W.T., Jackman M.R., Bizeau M.E., Pagliassotti M.J., Hazel J.R. (2000). Hyperthermia impairs liver mitochondrial function in vitro. Am. J. Physiol. Regul. Integr. Comp. Physiol..

[35] Justo R., Boada J., Frontera M., Oliver J., Bermudez J., Gianotti M. (2005). Gender dimorphism in rat liver mitochondrial oxidative metabolism and biogenesis. Am. J. Physiol. Cell Physiol..

[36] Hafner R.P., Brown G.C., Brand M.D. (1990). Analysis of the control of respiration rate, phosphorylation rate, proton leak rate and protonmotive force in isolated mitochondria using the ‘top-down’ approach of metabolic control theory. Eur. J. Biochem..

[37] Mildaziene V., Baniene R., Nauciene Z., Marcinkeviciute A., Morkuniene R., Borutaite V., Kholodenko B., Brown G.C. (1996). Ca^2+^ stimulates both the respiratory and phosphorylation subsystems in rat heart mitochondria. Biochem. J..

[38] Krishnamoorthy G., Hinkle P.C. (1984). Non-ohmic proton conductance of mitochondria and liposomes. Biochemistry.

[39] Lee W., Shin S., Cho S.S., Park J. (2000). Purification and characterization of glutamate dehydrogenase as another isoprotein binding to the membrane of rough endoplasmic reticulum. J. Cell Biochem..

[40] Ahn C.S., Metallo C.M. (2015). Mitochondria as biosynthetic factories for cancer proliferation. Cancer Metab..

[41] Friday E., Oliver R., Turturro F., Welbourne T., Canuto R.A. (2012). Role of Glutamate Dehydrogenase in Cancer Growth and Homeostasis. Dehydrogenases.

[42] Yang L., Moss T., Mangala L.S., Marini J., Zhao H., Wahlig S., Armaiz-Pena G., Jiang D., Achreja A., Win J. (2014). Metabolic shifts toward glutamine regulate tumor growth, invasion and bioenergetics in ovarian cancer. Mol. Syst. Biol..

[43] Jin L., Li D., Alesi G., Fan J., Kang H., Lu Z., Boggon T.J., Jin P., Yi H., Wright E.R. (2015). Glutamate Dehydrogenase 1 Signals through Antioxidant Glutathione Peroxidase 1 to Regulate Redox Homeostasis and Tumor Growth. Cancer Cell.

[44] Spinelli J.B., Yoon H., Ringel A.E., Jeanfavre S., Clish C.B., Haigis M.C. (2017). Metabolic recycling of ammonia via glutamate dehydrogenase supports breast cancer biomass. Science.

[45] Zukiene R., Nauciene Z., Ciapaite J., Mildaziene V. (2010). Acute temperature resistance threshold in heart mitochondria: Febrile temperature activates function but exceeding it collapses the membrane barrier. Int. J. Hyperth..

[46] Mármol F., Sanchez J., Lopez D., Martinez N., Xaus C., Peralta C., Roselló-Catafau J., Mitjavila M.T., Puig-Perellada P. (2010). Role of oxidative stress and adenosine nucleotides in the liver of aging rats. Physiol. Res..

[47] Muñiz P., Barchino M.J.G., Iradi A., Mahiques E., Marco V., Oliva M.R., Sáez G.T. (2000). Age-related changes of liver antioxidant enzymes and 8-hydroxy-2′-deoxyguanosine during fetal-neonate transition and early rat development. IUBMB Life.

